# Exploring the Potential of Stem Cells: A Systematic Review on Cellular Therapy for Sensorineural Hearing Loss

**DOI:** 10.7759/cureus.77286

**Published:** 2025-01-11

**Authors:** Maria A Henao Rincón, Edder A Pulido Arias, Catalina Pachon Rojas, Alejandro Gonzalez Orozco, Catherine Bolaños Lopez, Valeria Arango García, Alejandro Uribe Escobar, Carlos A Velez Duncan

**Affiliations:** 1 Otolaryngology, Universidad de Cartagena, Cartagena, COL

**Keywords:** bilateral hearing loss, cellular therapy, hearing sciences, sensorineural hearing loss, stem cells

## Abstract

Sensorineural hearing loss affects a significant portion of the global population, with its prevalence projected to rise sharply in the coming years. Most cases involve the degeneration of hair cells and spiral ganglion neurons within the inner ear, and current therapeutic options for hearing rehabilitation offer limited efficacy with variable outcomes among patients. This systematic review evaluates the existing evidence on stem cell therapy as an intervention for hearing loss, focusing on its impact on hearing restoration, quality of life, and safety. A thorough search of electronic databases and clinical trial registries identified randomized and quasi-randomized studies on this topic. Eight studies met the inclusion criteria, investigating various types of stem cells such as embryonic, umbilical cord, and inner ear cells administered intravenously or directly into the inner ear. Most studies used animal models to simulate hearing loss, while one was conducted in humans. Findings on hearing improvement were mixed, with some studies reporting significant improvements in hearing thresholds and others showing no effect. The safety of stem cell therapy was assessed in a single human study, which noted no significant adverse effects. While the results indicate potential therapeutic value, further human studies with standardized protocols and larger sample sizes are necessary to clarify the safety and effectiveness of stem cell therapy for sensorineural hearing loss.

## Introduction and background

Hearing loss is a condition that affects around 466 million people worldwide (432 million adults and 34 million children), according to the WHO [1,2], corresponding to approximately 15% of the general population [3]. It is estimated that by 2050, 1 in 10 people will suffer from hearing loss, representing more than 900 million individuals, and of these, over 72% will be over 65 years old [1]. The majority of cases are sensorineural hearing loss, characterized by the loss of hair cells at the cochlear level and variable degrees of spiral ganglion neuron loss [4].

Various genetic and environmental factors are implicated in the development of sensorineural hearing loss, such as mutations, viral infections, autoimmunity, chronic noise exposure, aging, ototoxic medications, and other factors that lead to similar degenerative consequences [5]. Permanent deafness results from the inability of the cochlea to regenerate cochlear hair cells, unlike vestibular hair cells, which can regenerate to a limited extent [6,7].

In humans, there is no endogenous cellular regeneration in the inner ear, nor is there an exogenous therapy that allows for the replacement of damaged cells. Currently, rehabilitation relies on hearing prostheses such as hearing aids and cochlear implants [4,8]. These devices yield variable results among patients, with limitations in hearing discrimination and a limited lifespan. The available technology is currently limited by the functional capacity of the remaining spiral ganglion neurons. More recently, therapies based on cellular regeneration with the transplantation of different types of stem cells, such as mesenchymal stem cells, embryonic stem cells, and induced pluripotent stem cells (IPSCs), have been under investigation to replace damaged cells and restore hearing function [3,7]. This aims to increase synapses, cellular differentiation, and proliferation, which is not achievable with the current hearing rehabilitation devices [3].

There is extensive information on the pathophysiology of the inner ear in many diseases, allowing for an increased possibility of implementing appropriate therapies for managing hearing loss. The typically late onset of hearing disability allows for a window of time between genetic diagnosis and symptom manifestation, enabling preventive and protective treatments to be administered for sensorineural hearing loss [3,4,7]. Advances in genetics and molecular targeted therapy have led to isolated studies on the use of stem cells in relation to hearing loss; however, these studies are methodologically limited and highly variable. This systematic literature review aims to evaluate the available evidence of stem cell therapy in hearing loss, specifically its impact on hearing, quality of life, and the safety of this therapy.

## Review

Methods

This systematic review was conducted in accordance with the Preferred Reporting Items for Systematic Reviews and Meta-Analyses (PRISMA) guidelines and the Cochrane Collaboration's standards for reporting systematic reviews and meta-analyses. Additionally, we ensured the absence of duplicates by thoroughly reviewing previously registered systematic reviews. Randomized and quasi-randomized clinical trials were included if they involved stem cell therapy for sensorineural hearing loss and assessed its impact on hearing and safety. The studies considered included human and animal models published between January 2012 and July 2022 in English or Spanish. The therapy had to be administered via cochleostomy or intravenous infusion, with outcomes measuring either hearing improvement or the presence of stem cells in the organ of Corti post-therapy. Interventions focused on the direct administration of stem cells to the inner ear or via intravenous infusion. The cells could originate from and be administered in both animal and human models. Studies focusing solely on in vitro proliferation and differentiation models of stem cells were excluded to avoid confounding effects.

Primary outcomes included hearing improvement, measured using objective audiologic tests such as auditory brainstem response and otoacoustic emissions (OAE). Secondary outcomes comprised the presence of stem cells in the inner ear, determined through histological analysis, cochlear immunohistochemistry, immunofluorescence, and polymerase chain reaction (PCR). Safety was assessed by identifying therapy-related side effects, while additional data, such as the origin and dose of administered stem cells, were also analyzed.

The identification of studies followed standard Cochrane criteria and methods. An electronic search was conducted across databases including PUBMED, EMBASE, Lilacs, and Scopus, utilizing search terms such as "mesenchymal," "stem," "cell," "inner ear," "mesenchymal stem cells," "hearing loss," and "hearing impairment." The specific search strategy included the Boolean string: ('mesenchymal' AND 'stem' AND 'cell'/exp OR 'stem' AND 'cell') AND ('inner' AND 'ear'/exp OR 'inner' AND 'ear') AND 'hearing' AND 'impairment.' Searches also included registered and completed clinical trials on clinicaltrials.gov and the International Standard Randomised Controlled Trial Number (ISRCTN) Registry.

Seven researchers independently and blindly screened the titles and abstracts retrieved during the bibliographic search using the Rayyan Intelligent Systematic Review tool. Studies meeting the inclusion criteria were subjected to a full analysis, and disagreements were resolved through discussion under the guidance of an expert otoneurologist.

Data extraction was conducted using a pre-designed Excel data extraction table, developed to ensure methodological rigor. The validity and reliability of the table were evaluated through a structured and systematic approach. Clear and detailed criteria for extracting each relevant variable were defined and consistently applied by the reviewers. To ensure the quality and accuracy of the extracted information, a second reviewer independently checked the data extraction table to identify potential errors or inconsistencies. In cases of discrepancies, these were resolved through discussion, and, if necessary, a third expert reviewer was consulted. This multi-step verification process, combined with standardized extraction criteria, ensured both the validity and reliability of the extracted data. Standard Cochrane methods, as described in the Cochrane Handbook for Systematic Reviews of Interventions, were applied throughout the analysis and data collection process.

Results

**Figure 1:**
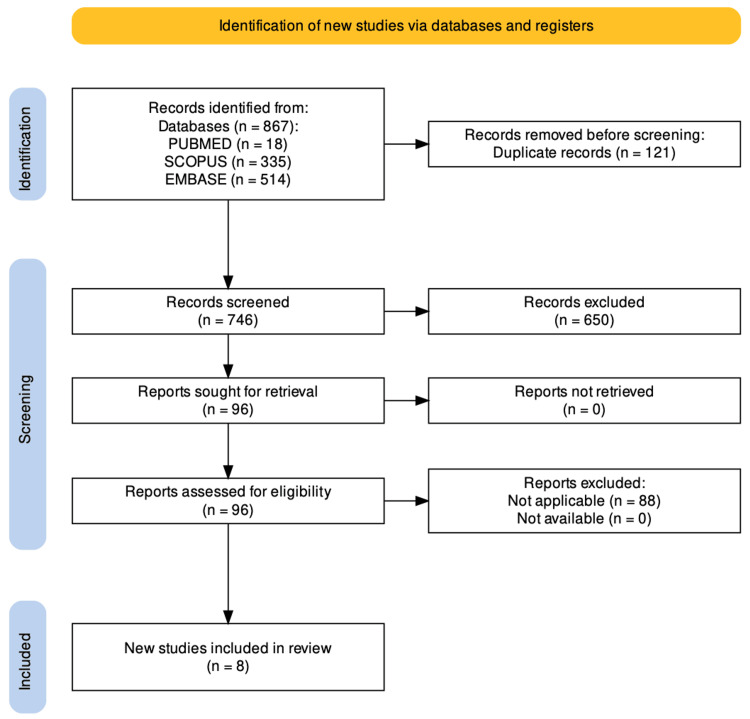
Flowchart

Through the database search, a total of 867 records were found. Ninety-six were selected for full-text review after removing duplicates, records older than 10 years, and articles written in languages other than English and Spanish, in addition to examining titles and abstracts. Eighty-eight of these were excluded based on exclusion criteria. Consequently, eight studies were included in the systematic review. This process can be found in more detail in Figure 1. The studies, their authors, the year of publication, the sample used, the species from which stem cells were obtained, where they were applied, as well as the method for inducing hearing loss and hearing measurement post-therapy were examined and compiled in Table 1.

**Table 1:** Studies included in the systematic review kg: kilograms; N: sample; mg: milligrams; mm: millimeter; microL: microliter; uM: micrometer; dB: decibel; PEA: auditory evoked potentials; OAS: otoacoustic emissions; ABR: auditory brainstem response; PEATC: brainstem auditory evoked potentials

Study	Country	Sample	Species/organ/tissue from which stem cells were obtained	Method used to induce damage to hair cells	Method of hearing evaluation	Site of stem cell implantation	Dose of transplanted stem cells	Results	
									1 [9]	Egypt	N = 10 males from which cells were extracted and 60 females, to which 4,000,000 cells were injected in 0.5 mL	Species: guinea pig (males). Organ: harderian gland (orbit)	Intraperitoneal injection of carboplatin (24 mg/kg) + 2nd dose at 2 days	Preyer's auricular reflex	Intravenous (guinea pig auricular veins)	4,000,000 cells in 0.5 mL	Hearing improvement after 3 weeks of transplant (80% showed positive reflexes).	
																		2 [10]	United States of America	N = 11 children who received between 8,000,000-30,000,000 cells/kg	Species: human. Organ/tissue: human umbilical cord blood	No induced damage, patients with acquired sensorineural hearing loss	OAE, ABR, audiometry, impedance	Intravenous	8,000,000-30,000,000 cells/kg	Safety evaluation in relation to adverse effects with intravenous injection, determining it to be safe. PEATC improved in 5 patients per month (p > 0.05). Improvement was permanent during follow-up. One patient worsened.	
3 [11]	South Korea	N = 21 male mice	Species: mouse. Organ/tissue: embryonic cells	Ouabain (gelfoam impregnated with 5 microliters of 1 mM solution) Kanamycin (gelfoam impregnated with 5 microL of 150 mg/kg solution)	PEA at 2 weeks in ouabain PEA at 2, 4, and 8 weeks in kanamycin	Tympanic scale (through round window)	3 uL (20,000 cells/uL)	No auditory improvement found between 2 and 8 weeks. Stem cell survival was observed.																		
									4 [12]	South Korea	N = 15 guinea pigs Placentas = 13 women	Species: human. Organ/tissue: placenta	10% neomycin + 5 M ouabain octahydrate in middle ear (gel form)	PEA and OAE at 1, 3, and 5 weeks	Intravenous (guinea pigs)	10,000,000 cells in 100 uL	Increased threshold of potentials describing hearing increase, but also increased spiral ganglion cells in all turns. That is, cell recovery depends on the cochlear environment.										
																		5 [13]	Mexico	Not described	Species: human. Organ/tissue: human dermal fibroblasts	Amikacin 400 mg/kg/day for 15 days intramuscularly	ABR (day 0 of amikacin) on days 19, 23, and 33.	Tympanic scale in basal turn (via cochlestomy in guinea pigs)	10,000 cells in 4 uL of culture medium	Stem cell survival for 2 weeks and in all cochlear turns. Auditory results are not discussed or shown.	
6 [14]	Turkey	N = 35 female albino Wistar mice	Species: mouse. Organ/tissue: mouse embryonic fibroblasts	Daily intramuscular injection of amikacin (600 mg/kg) for 14 days	ABR at 4 weeks	Tympanic scale in basal turn (via cochleostomy)	30,000,000 cells in 10 uL	No hearing improvement, nor stem cell survival.																			
									7 [15]	South Korea	N = 30 Sprague- Dawley mice	Species: human. Organ/tissue: embryonic cells.	Broadband noise with 115 dB for 3 hours daily for 5 days	Auditory brainstem response (ABR) at 4, 8, 16, and 32 kHz. Evaluated at days 0-3 and then days 15-18.	Tail vein (mice)	500,000 cells in 250 uL	Evaluates the auditory protection that stem cells could provide against noise exposure. Despite being venous administration, cells were detected in the spiral ganglion and lung. Appears to attenuate ototoxicity with the use of stem cells.										
8 [16]	Brazil	N = 8 guinea pigs. Cavia porcellus	Species: mouse. Organ/tissue: mouse inner ear	Intratympanic injection of 10% neomycin 7 days prior	Brainstem auditory potentials at 2 weeks post-transplant	Tympanic scale in basal turn (via cochleostomy)	10,000 cells in 10 uL	Stem cells found in vestibular, middle, and tympanic scales. No auditory improvement occurred.										

In the eight clinical trials included, four obtained stem cells from human tissues [9-16], while the remaining four obtained stem cells from rodents [9,11,14,16]. Only one study analyzed human subjects [10], while the others were conducted in rodents. Regarding the tissues from which stem cells were extracted, three studies used mouse embryonic stem cells (mESCs) [11,14], one study utilized human embryonic stem cell-derived mesenchymal stem cells (hESC-MSCs) [10], one study used stem cells from the Harderian gland [9], one from umbilical cord [10], one from placenta [12], one from dermal fibroblast stem cells [13], and one directly from the inner ear [16]. The method used for differentiation into otic progenitor cells was adequately described in five of the clinical trials [9,11-14] and not included in the remaining three studies.

The routes of administration used for stem cell transplantation were intravenous in half of the studies [9,10,12,15] and via the round window membrane in the other half [11,13,14,16], with a highly heterogeneous dose in the number of transplanted cells, as described in Table 1. Different methods were used in the clinical trials to induce hearing loss in the subjects receiving stem cells. Six of them induced ototoxicity with drugs: Carboplatin [9], Kanamycin [11], Neomycin [12,16], or Amikacin [9,14]; while one study induced hearing loss by acoustic trauma [15]. In only one study, subjects had a previous diagnosis of acquired sensorineural hearing loss.

The presence of stem cells at the cochlear level was visualized using electron microscopy and immunohistochemistry in most trials [11-16], albeit for one study that used PCR [9], and the remaining study did not perform a histopathological study of the cochlea as it was conducted in humans [10]. The anatomical site where stem cells were located after transplantation was generally the organ of Corti and the spiral ganglion [11,14,16].

Assessment of hearing recovery after stem cell transplantation was performed in all studies. Abd El Raouf et al. found in the animal hearing loss group the absence of pinna and startle reflexes on the third day after receiving the second dose of Carboplatin. These absent reflexes lasted until the end of the experiment; however, the group treated with stem cells showed positive reflexes in 80% of the subjects after three weeks post-transplantation, indicating hearing restoration. The time frame of three weeks post-transplant and reflex reappearance describes the potential time it takes for transplanted stem cells to differentiate into support cells and help restore damaged hair cells or differentiate into any other damaged cochlear component [9].

Baumgartner et al. in their 2018 study conducted stem cell therapy extracted from the human umbilical cord (hUCB) as treatment for acquired sensorineural hearing loss in 11 children. During a 12-month follow-up with OAE and hearing brainstem responses (ABR), the study revealed that the ABR threshold improved by more than 5 dB in five patients. However, the overall effect of the treatment was statistically significant (p < 0.05) in ABR for only 3 of the 10 evaluated frequencies.

In the study by Kil et al., ABR and OAE were performed on the first, third, and fifth weeks post-transplantation of stem cells in the hearing loss group. Significant differences in ABR threshold were found compared to normal animals. In the stem cell-transplanted group, the ABR threshold showed improvement between the first and fifth week, and there was an increase in the number of spiral ganglion neurons with normal cochlear turn formation at the first, third, and fifth week [12].

Lopez-Juarez et al. measured hearing through ABR before ototoxic injection (day 0 of Amikacin), and on days 19 (4 days after finishing Amikacin treatment), 23, and 33 (4 and 14 days after stem cell transplantation, respectively). The measured hearing changes were not statistically significant between groups before and after cochleostomy after stem cell application [13].

Chang et al. used mice in their study and induced hearing loss with ouabain (acute group) and kanamycin (chronic group). Using mESC application, results revealed no hearing improvement in the acute group nor the chronic group after two weeks post-transplantation compared with control groups. In this study, mESC transplanted via the round window did not produce hearing improvement in acute or chronic hearing loss [11].

Gökcan et al. in 2016 investigated the effects of iPSCs on mouse embryonic fibroblasts. Wistar albino rats (WAR) underwent amikacin injection to induce hearing loss, and after four weeks, underwent ABR hearing testing, and subject groups underwent IPSC transplant. Results did not present statistically significant differences in hearing loss or changes, nor did they observe differentiated stem cells immunohistochemically in the cochlea [14].

Barboza et al. conducted a study with eight guinea pigs, in which they demonstrated that there was no statistically significant difference between ABR hearing thresholds before and after implantation of mouse post-natal inner ear stem/progenitor cells (mIESC), both in the study group and in the control group. The control group consisted of guinea pigs that underwent the same procedure for hearing loss induction with intratympanic neomycin but did not receive mIESC transplantation. They did find that intratympanic neomycin as an ototoxicity method was effective (p = 0.01) and that mIESCs have stem cell properties and integrate into the basal and middle turns of the cochlea [16].

In the study by Kim et al., the injection of hESC-MSCs before the traumatic acoustic hearing loss was induced, and hearing ABR thresholds were measured at different frequencies. Results revealed a protective effect in the stem-cell injection group, with lower thresholds at 4, 8, and 16 kHz compared to the control group. Likewise, transplanted MSCs were present in the spiral ganglion as well as lung tissue.

Key findings

A significant proportion of the world's population has a hearing disability secondary to a cochlear dysfunction [17]. Unfortunately, the mammalian auditory system does not have the capacity to restore damaged hair cells, unlike other non-mammalian species [7,17]. Current management therapies for hearing loss have variable success [18], which has led in recent years to the development of new therapies, such as the use of stem cells, as proposed by Ibekwe et al. in their systematic review a decade ago [19]. Chorath et al. carried out a systematic review in 2020 that evaluated nine animal studies using xenogeneic stem cells derived from bone marrow or fetal tissue, revealing improvement in auditory function through an ABR threshold of 15.22 dB (p = 0.005), as well as in distortion product otoacoustic emission (DPOAE) improvement by a mean difference of 9.10 (p < 0.0001). These results revealed a superiority of extracochlear administration of stem cells compared to intracochlear injection [20]. Despite the promising progress in the field of stem cells and hearing loss, there is a broad absence of large systematic reviews that allow us to evaluate the effectiveness and safety of this therapy. Until the date of this manuscript, only two systematic reviews were found that included animal studies [19,20] only one of these objectively assessed hearing gain [20]. However, the safety of the therapy was not studied in the different articles. There is an ongoing interest in stem cell therapy and hearing loss, yet there are not enough studies to conclude or dictate a therapy based on their findings [19].

Understanding that hearing disability is an increasingly common problem in the population, a disabling condition that significantly affects the patient's quality of life [16,21,22], and an increase in health care costs, hence why the interest remains high for new therapies [23]. This systematic review demonstrates that there are a limited number of studies investigating the use of stem cells for the treatment of hearing loss. Eight studies were included, of which four used human stem cells, while the remaining four used stem cells from animal models. It is important to note that to date in this manuscript, only one study evaluated outcomes in humans [10].

Hearing Results

Hearing evaluation before and after stem cell transplantation was performed in all studies using different methods [9-16], only one tested it subjectively [9]. Three studies reported an improvement in hearing thresholds after stem cell treatment, while four studies showed no improvement. Despite the diversity of the tests used in the different studies, it did not affect the evaluation of hearing loss either before or after the administration of stem cell therapy.

The results were discrepant. In the Abd El Raouf et al. study, the stem cell-treated group showed improved hearing, as indicated by the restoration of auditory reflexes after three weeks of stem cell therapy [9]. Baumgartner et al. reported that ABR thresholds improved in five patients and that the overall treatment effect was statistically significant for 3 of the 10 frequencies tested. Furthermore, wave V latency improved after treatment with hUCB blood stem cells [10]. Kil et al. found significant differences in the ABR threshold between the group with hearing loss and the group transplanted with stem cells [12]. The study by Chang et al. reported that there was no improvement in hearing threshold after stem cell transplantation [11]. Similarly, Gökcan et al. found that post-injection hearing thresholds showed no statistically significant differences between the study groups, and no immunohistochemically differentiated stem cells were observed [14]. In this sense, it is worth highlighting that although the degree of hearing recovery was minimal in most studies, it is considered promising results.

Measuring Hearing Outcomes

All studies measured therapeutic results. In two of the studies, the auditory result was measured by OAE, one associated with audiometry [10] and another with brainstem auditory potentials [12]. In five studies they used potentials only [11,13-16] and one used the Preyer pinna reflex [9]. In three of the trials, serial auditory measurements were performed [11,13,14]. In three studies, improvement in hearing thresholds was found after treatment [9,10,12] while in four studies there was no improvement [11,13,14,16]. One of the studies did not aim to measure hearing improvement but rather non-hearing loss (protection) in subjects exposed to acoustic trauma [15].

Security in Administration

The study by Baumgartner et al. was the only one in humans that evaluated safety, monitoring toxicity through hemodynamic assessment, PO2, chest X-ray, blood count, kidney and liver function, and neurological examination, being normal in all patients. Clinical trials are needed that specifically evaluate the safety of this therapy in humans in the short, medium, and long term [10].

Hearing Loss Induction Method

The methods used to induce hearing loss in subjects receiving stem cells also varied, including ototoxicity with carboplatin, kanamycin, neomycin, and amikacin; acoustic trauma; and previous diagnosis of acquired sensorineural hearing loss.

There are very few studies on the use of stem cells as a preventive therapy against harmful auditory stimuli. Lopez-Juarez et al. found that stem cell therapy was able to prevent hearing loss in subjects exposed to amikacin [13]. In turn, Kim et al. reported that noise-exposed rats transplanted with stem cells had lower hearing thresholds and less outer hair cell loss than noise-exposed control rats [15].

Evaluation of the Presence of Stem Cells in the Inner Ear

The method used to evaluate the presence of stem cells at the cochlear level was very versatile. However, it did not affect the evaluation of hearing ability either before or after the administration of stem cell therapy.

Type of Stem Cells and Route of Administration

Stem cells were extracted from various tissues, including embryonic cells, umbilical cords, placentas, dermal fibroblast stem cells, and inner ear stem cells. One of the main limitations of this therapy is the difficulty in the differentiation of stem cells into ciliated cells. The heterogeneity of the studies in terms of origin and methods used to induce their differentiation [10,16] does not permit the establishment of a standard model. Even as mentioned by Devarajan et al., in their study, the use of gene therapy, viral vectors, and tissue engineering are proposed as promising technologies associated with the use of stem cells to promote cellular differentiation [21]. Regarding the route of administration, the intravenous route predominates, as well as directly at the level of the scala tympani, the latter being only tested in animal models given the risk of inducing greater hearing loss, without demonstrating superiority in terms of the migration capacity of stem cells to the inner ear compared to the intravenous route [11,13,14,16]. Only in the study by Baumgartner et al. was the intravenous route of administration used specifically in humans, without showing significant side effects. Clinical trials are needed to evaluate this and other routes of administration specifically in humans.

Limitations and bias assessment

This systematic review has several limitations, including its retrospective nature, the variability of studies due to stem cell therapy still being under investigation, and the high heterogeneity in methodologies. The limited data available on this topic highlights gaps in research on therapies for deafness worldwide. Furthermore, reporting biases may have influenced the synthesis due to differences in study designs, measurements, and methodologies across the included research. These biases could lead to an overestimation of the efficacy of stem cell therapy for sensorineural hearing loss. The variability in cell types, dosages, administration routes, and outcome measurement methods, along with the reliance on preclinical animal model data and the lack of standardized methodologies, introduces risks of inconsistent reporting and incomplete analysis. Addressing these limitations in future studies is crucial to advancing the implementation of effective treatments.

## Conclusions

This systematic review evaluated the use of stem cells for the treatment of sensorineural hearing loss. Although the results are limited and heterogeneous, our findings suggest that stem cell therapy may be a promising therapeutic tool for this condition. Some studies reported improvements in hearing thresholds, but the evidence remains inconclusive due to the diversity of methodologies and limited human studies. More research is needed, particularly larger-scale human trials with standardized protocols, to confirm the effectiveness, safety, and short-, medium-, and long-term effects of stem cell therapy. Overall, further studies are essential to fully understand the potential of stem cell transplantation to improve hearing and optimize the procedure for clinical use.
